# 

**Published:** 1889-04

**Authors:** 


### Society Meetings.

The Alumni Association of the Medical Department of Niagara University will hold its fourth annual meeting, Tuesday, April 9, 1889, commencing at eleven A. M., at the college building, Ellicott street. The programme includes the Address of Welcome, by Prof. A. E. Persons; the President's Annual Address, by Dr. C. C. Frederick; business session and election of officers. The afternoon session, at three o'clock, will be held in the Lecture Room Y. M. C. A. Building, at which the following papers will be read: Acute Infectious Osteo-Myelitis, by Herman Mynter, M. D., Buffalo, Professor of Surgery, Medical Department, Niagara University; The Principles of Treatment of Pulmonary Tuberculosis as based upon its Etiology and Pathology, by Hermann M. Biggs, M. D., New York, Demonstrator of Anatomy and Instructor in the Carnegie Laboratory, Bellevue Hospital Medical College; The Gynecic Element in Psychiatry, by Charles A. L. Reed, M. D., Cincinnati, Professor of the Surgical Diseases of Women, Cincinnati College of Medicine, Professor of Gynecology, Women's Medical College.

---

The Commencement Exercises of the Medical Department of Niagara University will be held Tuesday evening, April 9, 1889, at Association Hall, Y. M. C. A. Building. After the conferring of degrees, an address will be given by R. Stansbury Sutton, M.D., L.L.D., of Pittsburgh, Pa. The annual banquet will take place afterward at the Genesee. The medical profession and others interested are invited to the afternoon session of the Alumni Association, and the general public is also cordially invited to attend the commencement exercises in the evening.

---

The Annual Meeting of the National Association of Railway Surgeons will beheld at St. Louis, Mo., on Thursday and Friday, May 2 and 3, 1889. The prospects are that this will be one of the largest gatherings of medical men ever assembled in this country. Dr. W. B. Outten, of St. Louis, is the chairman of the committee of arrangements, and everything will be complete for the accommodation of the Surgeons. Any information desired can be had by addressing the Secretary, C. B. Stemen, M. D., Fort Wayne, Indiana.

---

The American Medical Association will hold its fortieth annual meeting in Newport, R. I., commencing Thursday, June 25, 1889, and continuing four days. From the indications on all sides, this promises to be one of the most interesting meetings, from a scientific point of view, that the Association has held in some years.

---

The Buffalo Medical and Surgical Association will hold its regular monthly meeting at Mechanics' Institute, No. 9 W. Mohawk street, Tuesday, April 2, 1889, at 8:30. Election of officers.

International Congress of Hydrology and Climatology.—The second session of this congress will be held in Paris, October 3–10, 1889. Scientific societies and associations, and the savants of France and foreign nations, are invited to participate in this international re-union. The fee for membership—twenty francs—entitles the holder of a ticket to admission to the exposition.

ANNOUNCEMENT.—J. B. Lippincott & Co., publishers, announce the Cyclopedia of the Diseases of Children, Medical and Surgical, as now ready, and are canvassing this city for subscribers. This most complete work by American, British, and Canadian authors, edited by John M. Keating, M. D., will, no doubt, find ready sale, as it covers an unique field in the cyclopedic literature of medicine. It is offered complete in four handsome imperial octavo volumes of about 800 pages each, and each volume is subdivided into from three to four parts, all profusely illustrated. Price per volume, cloth, $5.00; full sheep, $6.00; Half-Russia, $6.50. For sale by subscription only.

---

MESSRS. LEHN & FINK, 128 William street, New York, publishers of *Notes on New Remedies*, kindly offer to mail their journal regularly to any of our subscribers, without charge, who will request them to do so by postal, mentioning the fact that they are subscribers to the BUFFALO MEDICAL AND SURGICAL JOURNAL.

Now Ready.—Transactions of the American Association of Obstetricians and Gynecologists, Vol. I., 1888, supplied by William D. Dornan, publisher, North Seventh street, Philadelphia, Pa. Orders can be sent through any bookseller, or may be addressed to William Warren Potter, *Secretary*, 284 Franklin street, Buffalo, N. Y.

---

### BOOKS RECEIVED.

Wood's Medical and Surgical Monographs. Volume I., No. 3. March, 1889. Contents: Neurasthenia and its Treatment; by Dr. H. Von Ziemssen. Antipyresis and Antipyretic Methods of Treatment; by Dr. H. Von Ziemssen. The Tongue as an Indication of Disease; by Dr. W. H. Dickinson. On the Treatment of Cystic Goitre; by T. M. Hovell, F. R. C. S. New Remedies, from 1878 to 1888; by Dr. C. Cauquil. Index for Volume I. William Wood & Co., publishers, 56 and 58 Lafayette place, New York.

L'Enseignement et L'Organization de L'Art Dentaire aux Etats-Unis. Rapport adresse a Monsieur le Ministre de L'Instruction Publique; par Le Dr. Kuhn. Paris: Octave Doin, Editeur. 8 Place de L'Odeon. 1888.

The Psychic Life of Micro-Organisms. A study in experimental Psychology. By Alfred Binet. Translated from the French by Thomas McCormack. With a preface by the author, written especially for the American edition. Chicago: The Open Court Publishing Company. 1889.

Transactions of the Texas State Medical Association, Twentieth Annual Session, held at Armory Hall, Galveston, Texas, April 24, 25, 26 and 27, 1888. Austin, Texas: Printed for the Texas State Medical Association. Eugene Von Boeckmann, Steam Printer. 1888.

A Manual of Instruction in the Principles of Prompt Aid to the Injured. Designed for Military and Civil Use. By Alvah H. Doty, M. D., Major and Surgeon, 9th Regt., N. G., S. N. Y.; Attending Surgeon to Bellevue Hospital Dispensary, New York. New York: D. Appleton & Co. London: Caxton House, Paternoster Square. 1889. $1.25.

The International Medical Annual and Practitioners' Index: A Work of Reference for Medical Practitioners. Seventh year. New York: E. B. Treat & Company, 771 Broadway. London: Paternoster Row. Chicago: 199 Clark street. 1889. $2.75.

Suggestive Therapeutics: A Treatise on the Nature and Uses of Hypnotism. By H. Bernheim, M. D., Professor in the Faculty of Medicine, at Nancy. Translated from the second and revised edition by Christian A. Herter, M. D., of New York. New York and London: G. P. Putnam's Sons. The Knickerbocker Press. 1889. $3.50.

A Hand-book of Therapeutics. By Sydney Ringer, M. D., Professor of the Principles and Practice of Medicine in University College; Physician to University College Hospital. Twelfth edition. New York: William Wood & Co., 56 and 58 Lafayette place. 1889.

Kirke's Hand-book of Physiology.

Hand-book of Physiology. By W. Morrant Baker, F. R. C. S., Surgeon to St. Bartholomew's Hospital; Member of the Court of Examiners of the Royal College of Surgeons; Examiner in Surgery in the University of London and at the Royal College of Physicians; late Lecturer on Physiology at St. Bartholomew's Hospital, and Vincent Dormer Harris, M. D., London, Fellow of the Royal College of Physicians; Examiner in Elementary Physiology at the Conjoint Board of the Royal College of Physicians and Surgeons; Demonstrator of Physiology at St. Bartholomew's Hospital; Physician to the Victoria Park Hospital for Diseases of the Chest. Twelfth edition. Re-arranged, revised, and re-written, and with 500 illustrations. New York: William Wood & Co., 56 and 58 Lafayette place. 1889.

Sixteenth Annual Report of the State Commissioner in Lunacy, for the Year 1888. Transmitted to the Legislature, January 15, 1889. Albany: The Troy Press Company, printers. 1889.

Lectures on Nervous Diseases, from the standpoint of Cerebral and Spinal Localization, and the later Methods employed in the Diagnosis and Treatment of these Affections. By Ambrose L. Ranney, A. M., M. D., Professor of the Anatomy and Physiology of the Nervous System in the New York Post-Graduate Medical School and Hospital; Professor of Nervous and Mental Diseases in the Medical Department of the University of Vermont, etc. Profusely illustrated with original diagrams and sketches in color by the author, carefully-selected wood-cuts, and reproduced photographs of typical cases. Philadelphia: F. A. Davis, publisher. 1888.

Merck's Index of Fine Chemicals and Drugs, for the Materia Medica and the Arts, comprising a summary of whatever chemical products are to-day adjudged as being useful in either medicine or technology, with average values and synonyms affixed. A guide to the physician, apothecary, chemist, and dealer. By E. Merck. Darmstadt, New York and London. 1889.

Hand-book of the Diagnosis and Treatment of Skin Diseases. By Arthur Van Harlingen, M. D., Professor of Diseases of the Skin in the Philadelphia Polyclinic and College for Graduates in Medicine; Clinical Lecturer on Dermatology in the Jefferson Medical College. Second edition, enlarged and revised, with eight full-page plates and other illustrations. Philadelphia: P. Blakiston, Son & Co., 1012 Walnut street. 1889.

---

### PAMPHLETS RECEIVED.

Sub-acute Progressive Polymyostitis. By George W. Jacoby, M. D. New York: Reprint from the *Journal of Nervous and Mental Diseases*, November, 1888.

Pressure Forceps *versus* The Ligature and the Suture in Vaginal Hysterectomy. By E. C. Dudley, M. D., Chicago. Reprint from Vol. XIII., Gynecological Transactions. 1888.

Yellow Fever: Absolute Protection Secured by Scientific Quarantine. By Welford Nelson, C. M., M. D. New York. 1889.

The Sanitary and Economic Disposal of Garbage and Refuse by the Merz Method. "The Merz Universal Extractor & Construction Company," No. 60 Pearl street. Buffalo: Peter Paul & Bro., printers and binders.

Twenty-five Erhaltende Kaiserschnitte und die Stellung der Sectio Cæsarea zur Perforation. Von Prof. Dr. Leopold. Sonderabdruck aus *Archiv. für Genäkologie.* Bund XXXIV. Heft 2. Leipzig. 1889.

On the Relation of the Nasal and Neurotic Factors in the Etiology of Asthma. By F. H. Bosworth, M. D., E. L. Shurley, M. D., W. H. Daly, M. D., and Andrew H. Smith, M. D. Reprint from the *New York Medical Journal* for January 19, 1889.

On Some Mild Measures in the Treatment of Intra-Nasal Hypertrophies and Inflammations. By W. H. Daly, M. D., Pittsburgh, Pa. Reprint from the *Medical and Surgical Reporter* for November 17, 1888.

Periodicals, Transactions, and Reports in the New York Academy of Medicine. Part I: United States and Great Britain. By John S. Browne, Esq., Librarian. 1889.

Proceedings of the Department of Superintendence of the National Educational Association at its meeting in Washington, February 14–16, 1888. From the Bureau of Education.

The One Hundred and Eighteenth Annual Report of the State of the New York Hospital and Bloomingdale Asylum for the Year 1888. From Hon. Leroy Andrus, Member of Assembly.

Eleventh Annual Report of the Presbyterian Eye, Ear and Throat Charity Hospital, 1007 East Baltimore street, Baltimore, Md. From Dr. Julian J. Chisolm.

Annual Report of the Morse Dispensary of Cooper Medical College, San Francisco, Cal., for 1888.

Eighteenth Annual Report of the Managers of the Buffalo State Asylum for the Insane for the Year 1888.

Annual Commencement of Cooper Medical College, San Francisco, Cal. Session of 1889.

Health Reports and Bulletins: Tennessee State Board of Health Bulletins. February 15 and March 15, 1889.

Newport, R. I.: Report of Deaths and Contagious Diseases for the Month of February, 1889.

Michigan State Board of Health Bulletin: "Health in Michigan, February, 1889."

New York State Board of Health: Monthly Bulletin for January, 1889.

Weekly Abstract of Sanitary Reports U. S. Marine Hospital Service. Vol. IV., Abstracts Nos. 8, 9, 10, 11 and 12.

---

### AMONG OUR EXCHANGES.

Among the new journals which have appeared recently, we note the *North American Practitioner* the journal of the Post-Graduate Medical School, of Chicago, issued monthly and edited by Bayard Holmes, M. D., editor-in-chief, and Junius C. Hoag, M. D., associate editor, and published by Chas. Truax & Co.

The *Alabama Medical and Surgical Age*, a monthly journal of medical and surgical science, is edited by John C. LeGrand, and published at Anniston, Ala.

*Journal of the Respiratory Organs*, edited by J. Mount Bleyer, M. D., who announces seventeen collaborators, residing in this country and Europe. It is published in New York by Napoleon Thompson, 51 and 53 Maiden Lane.

The *Pacific Medical Journal* has thus simplified its title, and will be edited and published hereafter by D. A. Hodghead, A. M., M. D., 522 California street, San Francisco, California.

Dr. J. J. Mulheron has resigned as editor of the *Medical Age*, to devote his entire time to teaching and practicing his profession. He is succeeded by B.W. Parmer, M. D.

Dr. Virgil O. Hardon assumes the editorial management of the *Atlanta Medical and Surgical Journal*. We extend our congratulation to our above mentioned contemporary.

The *Chicago Law Times*, a quarterly journal, containing a department of medical jurisprudence, is edited by Catharine V. Waite, and published by the Waite Publishing Co., 96 and 97 Ashland block, Chicago.

